# 4D printed origami-inspired accordion, Kresling and Yoshimura tubes

**DOI:** 10.1177/1045389X231181940

**Published:** 2023-06-21

**Authors:** Anastasia L Wickeler, Kyra McLellan, Yu-Chen Sun, Hani E. Naguib

**Affiliations:** 1 Department of Mechanical and Industrial Engineering, University of Toronto, Toronto, ON, Canada; 2 Department of Materials Science and Engineering, University of Toronto, Toronto, ON, Canada; 3 Institute of Biomaterials and Biomedical Engineering, University of Toronto, Toronto, ON, Canada

**Keywords:** 4D printing, origami structure, accordion pattern, Yoshimura pattern, Kresling pattern

## Abstract

Applying tessellated origami patterns to the design of mechanical materials can enhance properties such as strength-to-weight ratio and impact absorption ability. Another advantage is the predictability of the deformation mechanics since origami materials typically deform through the folding and unfolding of their creases. This work focuses on creating 4D printed flexible tubular origami based on three different origami patterns: the accordion, the Kresling and the Yoshimura origami patterns, fabricated with a flexible polylactic acid (PLA) filament with heat-activated shape memory effect. The shape memory characteristics of the self-unfolding structures were then harnessed at 60°C, 75°C and 90°C. Due to differences in the folding patterns of each origami design, significant differences in behaviour were observed during shape programming and actuation. Among the three patterns, the accordion proved to be the most effective for actuation as the overall structure can be compressed following the folding crease lines. In comparison, the Kresling pattern exhibited cracking at crease locations during deformation, while the Yoshimura pattern buckled and did not fold as expected at the crease lines. To demonstrate a potential application, an accordion-patterned origami 4D printed tube for use in hand rehabilitation devices was designed and tested as a proof-of-concept prototype incorporating self-unfolding origami.

## 1. Introduction

Origami fold patterns with repeating unit cells can be used to inspire the design of advanced mechanical and structural materials. These patterns with repeating unit cells are commonly referred to as tessellated designs (Bertoldi et al., 2017). Changes to the unit cell pattern and dimensions can vary the materials’ overall mechanical properties. Parameterization of patterns and materials can be used to tune material properties of origami-inspired structures for specific application requirements (Reid et al., 2017). Properties that are commonly sought out in origami-inspired materials include bistability, tunable stiffness, enhanced energy absorption and negative Poisson’s ratio (Brunck et al., 2016; He et al., 2020; Liu et al., 2018a; Yasuda and Yang, 2015; Zhai et al., 2018).

There are various types of origami structures used to inspire mechanical materials. These include origami sheets with flat, and parallel, top and bottom surfaces, and curved origami structures that can be used as arced or tubular structures. Popular origami designs for flat core sandwich structures include the Miura-ori pattern and the Ron Resch pattern (Kshad et al., 2019; Lv et al., 2014). These are commonly studied for their high strength-to-weight ratios and their ability to absorb energy. Popular tubular and arced patterns include the Yoshimura, Kresling and accordion fold patterns (Jianguo et al., 2015; Kidambi and Wang, 2020; Kresling, 2020; Li et al., 2020; Masana and Daqaq, 2019). Tubular origami shapes are studied for a variety of applications. For example, Westra et al. (2021) used the Kresling and Yoshimura patterns to create bellows from thin-film polymers. Thin-film polymers typically have low fatigue life, and Westra et al. demonstrated that folding the film into an origami shape extends the fatigue life of the bellows. Kaufmann et al. (2022) utilized the bistability of the Kresling pattern to create a reconfigurable robotic arm with localized bending stiffness through parameterization and experimental validation.

Tubular origami can also be used to improve desired structural characteristics and it has been proposed that tubular structures can be utilized as energy absorbers due to their high specific energy absorption capability (Zhang et al., 2009). Recently, Li et al. (2019) reviewed the emergence of architected origami materials, which have unique and desirable properties such as nonlinear stiffness, impact absorption and multistability. The advances in crease design, mechanics modelling and scalable fabrication have led to the development of these materials. Filipov et al. (2016) reported the utilization of origami to create deployable, reconfigurable and unique three-dimensional structures by assembling thin sheets into origami tubes with polygonal cross-sections that can reconfigure into numerous geometries. The tubes satisfy the mathematical definitions for flat and rigid foldability and can follow non-linear curved lines when deployed. In another study, Filipov et al. (2015) introduced a new method of coupling origami tubes in a ‘zipper’ fashion to substantially increase system stiffness and permit only one flexible deployment mode. The resulting structure has an unusually large eigenvalue bandgap, representing the unique difference in stiffness between deformation modes. Yang et al. (2016) showed that Yoshimura-patterned brass tubes had lower peak crushing forces and the ability to increase energy absorption compared to conventional circular tubes. Liu et al. (2019) studied Miura-ori stiffened tubes compared to traditionally stiffened tubes and determined through experimentation that the Miura-ori stiffened tubes had higher load bearing capacity.

In addition, the unique behaviour of the origami structure can also be utilized for soft robotic actuator applications Pagano et al., 2017. For example, Zhang et al. (2021) described the design and implementation of a soft, earthworm-like robot that utilizes a non-rigid folding pattern of the Yoshimura structure to achieve 3-dimensional locomotion. Similarly, Kaufmann et al. (2022) reported the development of a new type of robotic arm utilizes Kresling origami-inspired design principles to achieve high levels of flexibility and manoeuvrability. Through extensive parametric analysis and experimental validation, the group determined the optimized Kresling pattern for constructing a prototype robotic arm consisting of three Kresling origami modules and the results showed that the robotic arm exhibited the desired reconfigurable articulation behaviour with consistent deformations. Validation of the mechanical properties of origami-inspired tubular structures through experimental testing shows the potential of how these materials can be applied in engineering applications, and the benefit of using these structures compared to more traditional materials.

The manufacturing of origami-inspired materials can be accomplished through a variety of means, including folding thin sheets, moulding and 3D printing. Origami-inspired materials that have been folded from thin sheets are typically referred to as foldcore structures (Fischer, 2015; Heimbs et al., 2010). The disadvantages of this technique include limitation in materials that can be used for this type of fabrication and residual stresses at crease locations where the folded material had to be plastically deformed to retain its origami shape. Moulding the structures overcomes the issue of residual stresses at crease locations; but may limit the number of configurations that can be tested due to the high cost of creating moulds (Kshad et al., 2017). 3D printing offers a viable and cost-effective solution in terms of a manufacturing method that can easily manufacture a parameterized set of origami designs.

3D printing is a rapidly progressing technology that is continually expanding its library of possible print materials. It includes materials such as metals, composites, ceramics, polymers, biomaterials and smart materials (Shahrubudin et al., 2019). Additively manufacturing smart materials is known as the process of 4D printing, as the stimuli-responsive aspect of the materials adds an extra dimension (Zhou et al., 2015). 4D printing of origami-inspired materials adds new possibilities for the prospects of origami-inspired materials for use in engineering applications (Langford et al., 2021; Tao et al., 2020; Zhang et al., 2018). For example, Liu et al. (2018b) 4D printed a Miura origami structure and measured the recovery force of the heat-activated structure for potential applications in space-saving actuation devices. The Miura-ori pattern was also previously studied by Kshad et al. (2017) as a shape memory origami design. The self-folding of the active Miura origami showed how thermal energy can be converted to mechanical work. Exploring the 4D printing of other origami patterns that fold into different shapes can help to create collapsible and foldable actuators that can be used in different applications based on the geometrical requirements of a given device.

This study will focus on creating novel 4D printed flexible PLA tubular structures for space-saving activation. Three tubular origami designs, the Yoshimura, Kresling and accordion fold patterns, will be assessed as possible candidates for heat activated unfolding origami structures. The various patterns will be tested using quasi-static compression testing to examine how the origami structures collapse when folded, and to test their strength. Ideally, the deformation should occur through folding at the origami creases to create a predictable and repeatable folding and unfolding scenario. The three patterns will then be tested as heat-activated, shape memory, self-unfolding, origami and their deformation and recovery will be tracked and compared to determine the suitability of the three designs for shape memory applications. The most effective origami pattern will then be used to test a proof-of-concept SMP hand rehabilitation prototype powered by the self-unfolding origami.

## 2. Origami designs and experimental tests and setup

### 2.1. Shape memory activation of origami tubes

**Figure 1.**
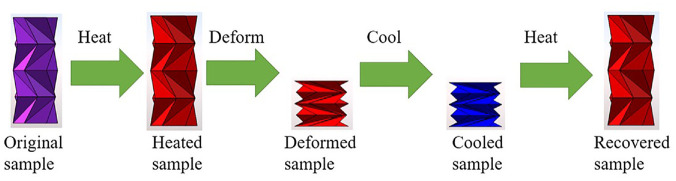
Shape memory cycle of heat activated 4D printed tubular structure.

The flexible PLA is a heat activated shape memory material. The activation cycle can be seen in Figure 1. The activation cycle starts with heating the sample above the material’s glass transition temperature (T_g_), then deforming the sample into its programmed shape and letting the sample cool. Once cooled, the deformed sample can then be reheated to recover its original shape.

During any given test, the shape programming temperature and activation temperature were the same. Two cycles of each test were performed in an oven at three different temperatures; 60°C, 75°C and 90°C. Two cameras were used in a stereovision setup to track the changes in the tubular origami structures during activation. The camera setup can be seen in the Supplemental Information Figure S1. Stereo vision uses synchronous images from two cameras capturing images of the same object to extract 3-dimensional data from the images. The digital image correlation was performed using the ‘Camera Calibration Toolbox for Matlab’ created by Jean-Yves Bouguet (Bouguet, 2015). The cameras were calibrated using 20 images of a checkerboard pattern, with known dimensions, at different angles. The camera calibration toolbox determines the focal length, principal point, skew and distortion of each camera and the rotational vector and translation vector of the right camera with respect to the left camera. The toolbox also includes a function that will output the 3D coordinates of a point visible in both cameras with respect to the cameras’ of a point when given the camera calibration parameters and the pixel coordinates of the location to be measured in the right and left images. Using a black pen, a point was drawn at the top and bottom of each activated sample. The height of the origami-inspired tubes was measured by determining the 3D coordinates of the top (x_1_, y_1_, z_1_) and bottom (x_2_, y_2_, z_2_) point, and calculating the distance between the points using equation (1).

(1) $$\left(\mathrm{distance}=\;\sqrt{{\left(x_{2}-x_{1}\right)}^{2}+{\left(y_{2}-y_{1}\right)}^{2}+{\left(z_{2}-z_{1}\right)}^{2}}\right)$$

The proof-of-concept rehabilitation application was also testing in the oven and measurements were taken with the stereovision setup. The results showed that the accordion pattern activated at 75°C was the most effective configuration, therefore this configuration was used in the application. A hinged hand model was 3D printed using rigid PLA, and the heat activated unfolding origami was printed as a curved accordion structure using the SMP flexible PLA. The programmed shape of the curved origami was set so that during the activation the finger transition from straight to curved. Points were marked in the centre of the hexagons of the accordion pattern so that the locations of the points can be measured using the stereovision camera setup.

### 2.2. Materials testing of flexible PLA filament

Differential scanning calorimetry (DSC, Q200, TA Instruments) tests were performed. The filament underwent a heat-cool-heat cycle. The experiment was performed at three different heating rates, 5°C/min, 10°C/min and 20°C/min, and the tests were carried out between 250°C and −50°C. Tests at different temperatures used different samples, and the second cycle of each test was graphed in the results section.

The thermogravimetric analyzer (TGA, Q50, TA Instruments) was used to analyse the thermal degradation of the filament. The experiment heated the samples from room temperature to 600°C. The test was performed at three different heating rates: 5°C/min, 10°C/min and 20°C/min.

To validate the mechanical properties, tensile tests were performed on the 3D printed flexible PLA according to the ASTM D638 standard (ASTM International, 2006). The tests were performed at a strain rate of 10 mm/min using type V style test specimens. Specimens were printed in two different print directions: horizontal and vertical. This allowed the direction-dependent material properties to be captured.

### 2.3. Origami-based tubular design details

**Figure 2.**
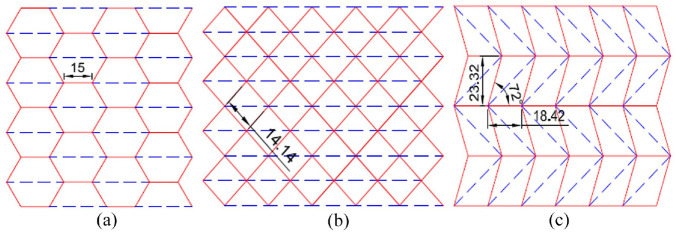
Fold patterns of the three origami designs used to create the tubular structures tested in this study. The red solid lines represent mountain folds, and the blue dashed lines represent valley folds. All dimensions are in millimetres. (a) The accordion pattern comprises of interlocking regular hexagons, outlined in red mountain folds. The regular hexagons are bisected horizontally through their centre with blue, dashed, valley folds. (b) The Yoshimura pattern is comprised of squares in a diamond orientation, which are outlined in red mountain folds. All the squares are the same size. The squares are bisected with horizontal lines though their centres with blue, dashed, valley lines. (c) The Kresling pattern is made with interlocking rhombuses. The rhombuses, outlined in red mountain fold lines, are mirrored along horizontal lines between rows so that the direction in which the rhombuses tilt vary between the rows. The outer dimensions of the rhombuses are all the same. Each rhombus is bisected by a blue dashed line alone their longest diagonal.

The three origami designs used to inspire the tubular shapes in this study are the accordion, Yoshimura and Kresling patterns. Images of the fold patterns used to create the shape of the outer surface of the tubular origami samples produced in this study can be seen in Figure 2, along with the pattern dimensions. The accordion design shown in Figure 2(a) is created with an interlocking series of regular hexagons folded as mountain folds, with an intersecting valley crease through the centre of each hexagon. The Yoshimura pattern shown in Figure 2(b) is comprised of squares in a diamond orientation that fold outwards in a mountain fold and are bisected by a valley fold line. Figure 2(c) shows the Kresling fold pattern. This design is made from a series of rhombuses with all the edges folded outwards in the mountain direction, and a line bisecting the rhombus that folds inwards with a valley fold. Images of the folded paper origami models for the patterns shown in Figure 2 can be seen in the Supplemental Information Figure S2.

Due to differences in their folding patterns, it is expected that the three origami structures would exhibit different deformability during compression and recovery behaviour. The accordion pattern is composed of parallel and connected mountain and valley folds, which can be compressed and expanded along their length. This creates a structure that can stretch and contract along its length, making it highly deformable under compression. In contrast, the Kresling pattern is made up of multiple interconnected and angled pleats that form a tubular structure. This structure can undergo reversible bistable deformation, allowing it to switch between a highly flexible state with low bending stiffness and a rigid state with high bending stiffness. This bistability allows the Kresling pattern to exhibit a wide range of deformations in different directions, as demonstrated in the literature (Kaufmann et al., 2022). Lastly, the Yoshimura origami pattern is formed by a repeated zigzag folding of an initially flat sheet, resulting in a set of parallelograms interconnected by hinges at the vertices. The interconnection between the parallelograms creates a saddle shape in the opposite direction of the folding. As a result, the Yoshimura pattern exhibits a relatively high level of mechanical stability due to its rigid and interconnected structure. However, this mechanical stability comes at the expense of reduced actuation capabilities, as the pattern is less able to transform between different configurations compared to the accordion and Kresling patterns when the folding pattern is oriented into a tubular structure.

The origami-based tubular design patterns have previously been studied as rigid tubes for structural purposes and as origami-inspired bellows folded out of thin films. Fabricating and characterizing the behaviours of these origami-inspired tubes from a flexible material is novel and it is unknown how these patterns will perform under quasi-static compression testing and as a heat activated unfolding origami shape memory structure. Testing and comparing all three patterns will enhance the understanding of how the fold patterns affect the behaviour of flexible tubular structures.

### 2.4. 3D printing of flexible origami tubes

**Figure 3.**
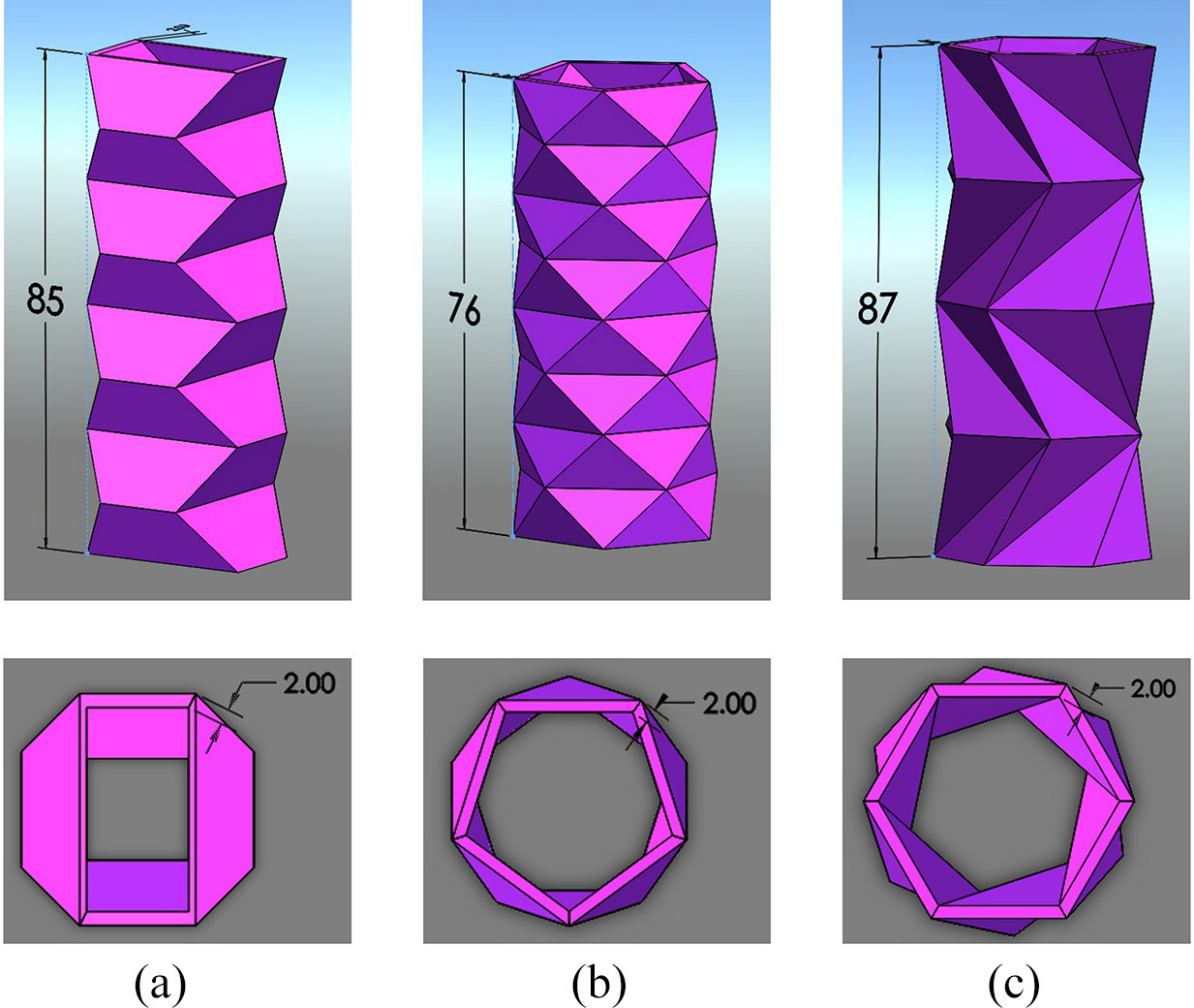
CAD models of the origami-inspired tubular structures that were 3D printed, quasi-static compression tested and shape memory activated as self-unfolding origami structures. All dimensions are in millimetres. The outer surfaces of the origami tubes were created based on the folding patterns and dimensions shown in Figure 2. (a) Accordion origami-inspired pattern; isometric and top view. (b) Yoshimura origami-inspired pattern; isometric and top view. (c) Kresling origami-inspired pattern; isometric and top view.

The samples produced in this study were made using a flexible PLA filament and were 3D printed using fuse deposition modelling (FDM) technology. Origami, which is typically folded from paper, has a negligible thickness. For the purposes of 3D printing these shapes using PLA, a thickness had to be added to the origami tubes. The origami fold patterns shown in Figure 2 were used as the dimensions for the outer surfaces of the tubes. The thickness at crease locations were made to be 2 mm, added on the inside of the tube and on a trajectory that points towards the centre axis of the tube. Faces were created on the inside of the tubes to connect all the thickened creases, and the areas between the inner and outer faces were filled to create a solid. Top views of the origami-inspired tubes are included in Figure 3 and show the thickness of the samples.

PLA filament was chosen because it is a bio-based and biodegradable polymer with shape memory properties (Huang et al., 2009). Most PLA filaments used in 3D printing are rigid; rigid materials are easier to print and less likely to jam in entry-level 3D printing machines. However, as technology advances with printer and materials alike, more material options are easily accessible. The flexible PLA filament used to create the origami-inspired tubular structures has a shore harness of 85A and is supplied by SPOOL3D. Origami structures tend to have many sharp corners and points in their structures. Using a flexible PLA to print the origami structures means that the edge crease areas of the origami will be softer and potentially safer in applications, such as rehabilitation devices, compared to a rigid origami structure. The flexible PLA also has a higher elongation until break compared to the rigid PLA. Compression testing the flexible origami structures will aid in predicting how the origami structures will fold during activation more effectively than a rigid material that is likely to crack under large strains. The 1.75 mm diameter flexible PLA filament was printed at a temperature of 205°C, with a print bed temperature of 50°C. The print speed was 40 mm/s, with a layer height of 0.18 mm and an infill density of 100%.

It should be noted that the mechanical performance of 3D printed parts can be significantly impacted by the specific print settings used, including printing speed and layer thickness (Doshi et al., 2022). Higher printing speeds can result in faster print times, but may also lead to reduced consistency and accuracy. Similarly, thicker layers can also result in faster print times, but as the layer thickness is decreased, the number of layers required for finishing the print increases, which results in increased fabrication time for the specimen. In our study, we used a specific set of print settings, including a moderate printing speed and layer thickness recommended by the printer’s manufacturer, to ensure a balance between print time and mechanical performance.

### 2.5. Compression testing of tubular designs

The three patterns of origami tubes were compression tested according to ASTM D1621. The samples were tested at a rate of 5 mm/min at room temperature condition and during testing, a camera was set to take pictures at a rate of 1 picture/s. The test setup can be seen in Supplemental Information Figure S3. These test images were used to create timelapse videos that show how the origami patterns deform as they are compressed at a constant rate. The deformation mechanics observed during the compression testing can help predict how the origami-inspired pattern will deform and deploy during the shape memory activation. The stress versus deformation curve data from the quasi-static compression test will also be useful for comparing the strength of the various designs when they are fabricated using a flexible PLA material.

## 3. Results and discussion

### 3.1. Flexible PLA material properties

**Figure 4.**
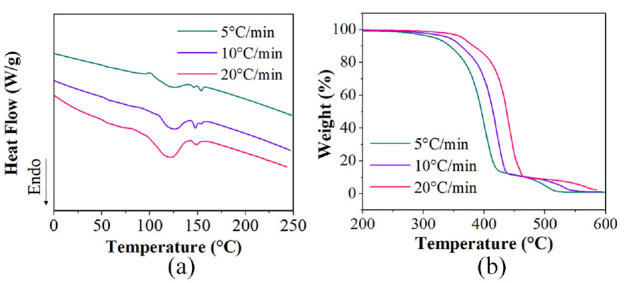
(a) DSC curve and (b) TGA curve for the flexible PLA filament taken at 5°C/min, 10°C/min and 20°C//min.

The TGA thermal degradation results can be seen in Figure 4(b). The initial degradation temperatures were 329°C, 344°C and 363°C for the 5°C/min, 10°C/min and 20°C/min tests respectively. This shows that at higher heat rates, the degradation temperature also increases. The flexible PLA filament exhibited a single step degradation for all three tests.

The DSC experiment was performed at three different heating temperatures, 5°C/min, 10°C/min and 20°C/min, for the flexible PLA 3D printing filament and the DSC curves can be seen in Figure 4(a). Analysis of the DSC curves was done to determine the glass transition temperature (T_g_), cold crystallization temperature (T_cc_), melting temperature (T_m_), enthalpy of cold crystallization (DH_cc_), enthalpy of melting (DH_m_) and percent crystallinity (Xc%). The percent crystallinity was calculated by subtracting the enthalpy of cold crystallization from the enthalpy of melting, then dividing that value by the enthalpy of fusion for perfectly crystalline PLA, 93.6 J/g (Huang et al., 2009). The results of the DSC curve analysis can be seen in Table 1.

**Table 1.** Thermal properties of flexible PLA at varied heating rates.

	T_g_ (°C)	T_cc_ (°C)	T_m_ (°C)	DH_cc_ (J/g)	DH_m_ (J/g)	Xc%
5°C/min	54.17	94.10	125.73	1.434	11.88	11.16
10°C/min	55.31	94.52	125.89	0.01243	10.29	10.98
20°C/min	53.17	95.41	122.37	0.05884	9.259	9.83

The DSC results show that the T_g_ ranges between 53°C and 55°C; therefore the three activation temperatures, 60°C, 75°C and 90°C, are all above T_g_, as they should be. The T_cc_ and T_m_ also show little difference at different heating rates, ranging between 94°C–95°C and 122°C–126°C respectively. The T_m_ shows that the chosen 3D printing extrusion temperature of 205°C was appropriate since it is higher than the melting temperature. The DH_cc_ and DH_m_ show a trend of decreasing with increased test temperature rates. The fact that the values of DH_cc_ and DH_m_ are different shows that the flexible PLA polymer is not amorphous. The calculated Xc% decreases as the heat rate increases, therefore the slower the polymer is being heated and cooled, the higher the amount of crystallinity observed in the sample.

**Figure 5.**
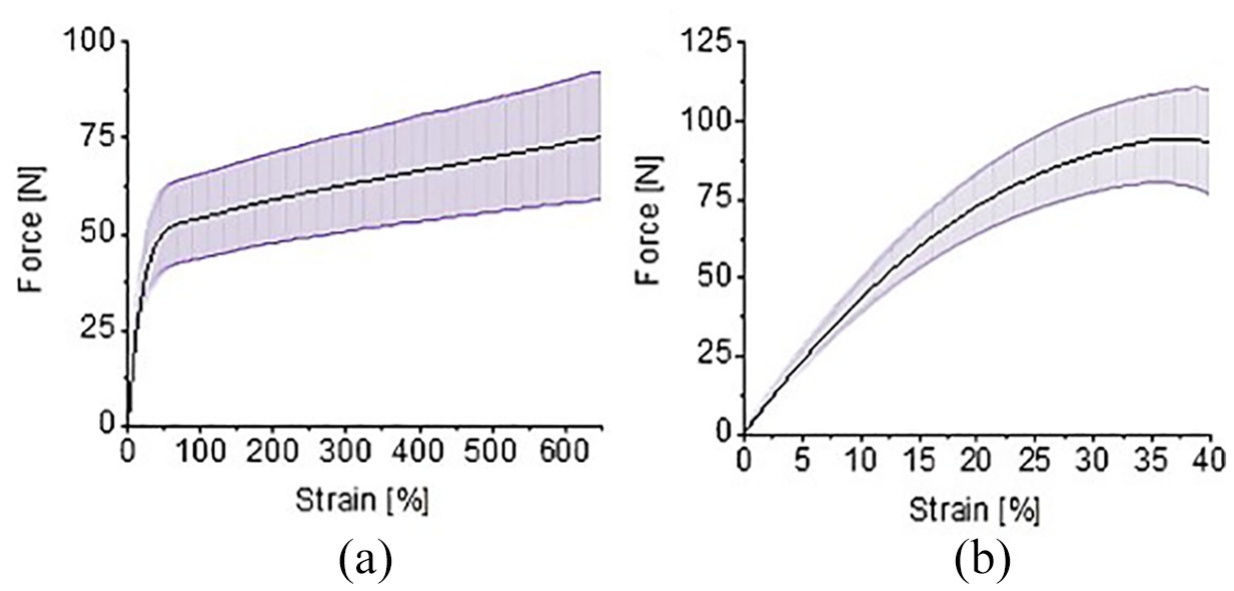
Tensile test results of flexible PLA: (a) printed horizontally and (b) printed vertically.

The results of the tensile tests can be seen in Figure 5. In the horizontal print direction, the specimens elongated to a strain of 650% and in the vertical print direction the specimens elongated to a strain of 40%. Tensile tests of 3D printed rigid PLA performed by the author in a previous study show a strain of approximately 25% in the horizontal direction and 4% in the vertical direction (Wickeler and Naguib, 2020). When comparing the rigid and flexible 3D printed PLA, the flexible print in the horizontal direction had an elongation 26 times longer than the rigid print in the same direction. The vertical flexible test elongated 10 times further than the rigid PLA. The differences in the strains between the two PLA materials shows that there is a significant difference in behaviour between the rigid and flexible PLA filaments under mechanical loads. The large strain percentages of the flexible PLA show that it can be considered an elastic material (Sun et al., 2017).

The vertical direction failed at a lower strain rate due to delamination between the print layers. In the horizontal print direction, the 3D printed material was being pulled along the length of filament, therefore the tensile properties were less reliant on the adhesion between layers and dependent on the intrinsic properties of the flexible PLA filament.

### 3.2. Compression testing of the origami-inspired tubes

**Figure 6.**
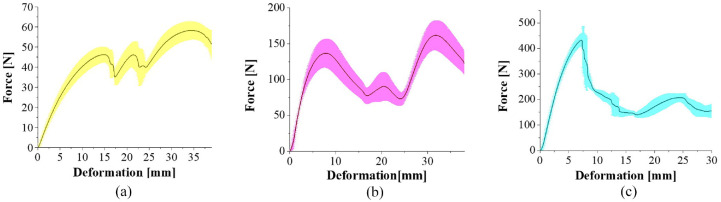
Force versus deformation plots of the three origami-inspired tubes: (a) accordion pattern, (b) Kresling pattern and (c) Yoshimura pattern.

The three origami-inspired tubular patterns were compression tested at a rate of 5 mm/min and the resulting force versus deformation compression graphs can be seen in Figure 6. Overall, the Yoshimura pattern was able to withstand the highest loads, followed by the Kresling pattern. The accordion pattern offered the least resistance to being folded. Looking at its force versus deformation graph, there are peaks and valleys in the curve. As the origami tubes are being compressed, the deformation in the tubes is concentrated at the crease areas. Once a series of creases passes a critical point, the required load to maintain a deformation of 5 mm/min decreased and hit a low point when the unit cell being folded finished folding. This process of multiple buckling of the rows of origami in the accordion and Kresling patterns accounts for the peaks and valleys observed in the force versus deformation graphs. Changes to the patterns, including varying the dimensions and number of unit cells in the tubular patterns may affect the peaks and valleys observed during compression testing, however parameterization of these patterns is outside the scope of this paper. The Yoshimura pattern did not follow this same trend, as it tended to buckle, and not fold at the creases like the accordion and Kresling patterns.

**Figure 7.**
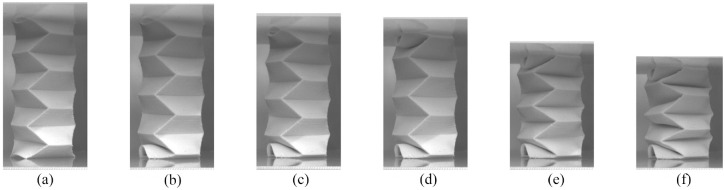
Compression progression of the flexible PLA accordion tubular origami pattern. (a) At 15.3 mm of deformation there is a peak in the applied load. After this point the bottom unit cell folds and the slope in the force-deformation curve becomes negative. (b) The bottom layer of the origami is completely folded. This occurs at a low point in the force-deformation curve. (c) At a deformation of 21.2 mm the accordion pattern experiences another force peak. Compared to the image at 17.0 mm deformation, the top unit cell has deformed slightly but has not completely folded yet. (d) This image is at a low force valley-point in the curve. This low point occurs as the top layer of the origami completely folds. (e) The final and highest force peak in the force-deformation cure occurs at 33.9 mm deformation. Here, all the remaining unfolded cells of the origami are being compressed. (f) As all the remaining unit cells in the origami are being folded, the amount of load required to deform the origami is decreasing.

Figure 7 shows the progression of how the accordion origami pattern folded during the compression testing. A video of the accordion compression test can be seen in the Supplemental Information Video S1. Figure 7(a) shows the tube at the first peak in the force versus deformation graph. There is deformation visible in the bending of the creases at the bottom section of the tube. The force-deformation curve had a negative slope between Figure 7(a) and (b). Looking at the images, it can be seen that the bottom row of the origami cells completely folded between the compression from 15.3 to 17.0 mm. This shows that once a fold starts to deform, it reaches a critical angle where the amount of force required to finish folding the given section starts to decrease. This same trend is visible between the images in Figure 7(c) and (d). The image in Figure 7(c) is taken at the second peak, and Figure 7(d) is taken at the second low point in the force-deformation curve. Between the deformation of 21.2 and 22.9 mm the force-deformation curve is negative again, showing that after a critical amount of deformation in the creases, the amount of load required to finish folding the top row of the origami decreases. The image in Figure 7(e) shows the accordion patterned tube at the final force peak before the centre rows of the origami pattern all start folding in Figure 7(f). Overall, the top and bottom rows of the origami tube were the first to fold. The origami faces in the centre area of the tube were all joined to adjacent faces through the creases. This seemed to strengthen the centre area of the tube since the creases in the centre of the tube were the last to deform and fold. At the top and bottom edges there are no adjoining faces, leaving open edges that are less able to resist deformation. Each section of creases (bottom row, top row and centre region), regardless of location, resisted deformation to a point where there is a peak in the force-deformation curve, which was followed by a low point in the force-deformation curve as the section finished folding.

**Figure 8.**
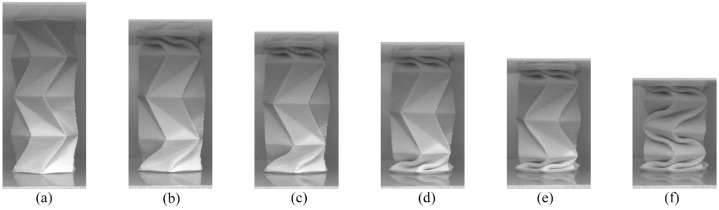
Compression progression of the flexible PLA Kresling tubular origami pattern. (a) At 7.8 mm deformation there is a peak in the Kresling force-deformation graph. After this point in the test, the applied load starts to decrease as the top and bottom rows continue to fold and twist. (b) The 17.3 mm deformation is at a valley point in the force-deformation curve. At this point the top row finished folding. (c) This image occurs at a slight peak in the force-deformation graph as the bottom row of the origami structure is being folded. (d) The force again hits a valley point as the bottom row of the origami finished folding at a deformation of 24.2 mm. (e) At 31.1 mm deformation there is another peak in the force-deformation curve as the centre rows of the Kresling pattern are starting to twist and deform. After this point the force required to continue folding the centre section of the Kresling pattern starts to decrease. (f) The folded Kresling pattern at a deformation of 42.4 mm.

The Kresling pattern deformation images can be seen in Figure 8. The video of the compression test can be seen in the Supplemental Information Video S2. The deformation in the Kresling pattern is similar to the accordion pattern in that the force-deformation curve also had a series of high and low points, and that the top and bottom rows of origami folded before the centre section. The image in Figure 8(a) was taken at the first peak and the image in Figure 8(b) was taken at the first valley in the force-deformation curve. The negative slope in the force-deformation curve between 7.8 and 17.3 mm deformation occurred as the top row in the origami pattern finished folding, showing that there is a critical point in the folding mechanism where the required folding force decreases, and the tube is less resistant to applied loads. After the top row finishes folding at a deformation of 17.3 mm, the bottom row is folded. Figure 8(c) was taken at the next peak location in the force-deformation curve, and Figure 8(d) was taken at the next low point. The peak at 19.9 mm deformation is lower than the peaks at 7.8 and 31.1 mm deformation. This is because when the top row of the structure is folded at 17.3 mm deformation, there is also deformation visible in the creases at the bottom row. The reason both the top and bottom rows of the Kresling origami were deforming simultaneously, which was not seen in the accordion compression testing, is that the Kresling pattern rotates in a slight twisting motion when it is folded. This twisting motion is visible when comparing the location of the horizontal creases and points as the compression test progresses. When the top row twisted while folding, it created a torque load in the tube that resulted in a similar rotation in the bottom row of origami cells. The rotation at the bottom of the tube caused the creases in the bottom row to start folding. The reason the peak in the force at 19.9 mm deformation is smaller than the peaks at 7.8 and 31.1 mm deformation is that the bottom row of the origami was already partially folded and needed only a small amount of additional force after the first valley point at the 17.3 mm deformation to continue folding easily. Figure 8(e) shows the structure at the last force-deformation peak and Figure 8(f) shows the structure when all the rows of the Kresling pattern are folded. Similar to the accordion pattern, the edge rows of origami folded first as the parts of the tubes with open outer edges were less able to resist the compression forces than the origami pattern in the centre of the tube.

**Figure 9.**
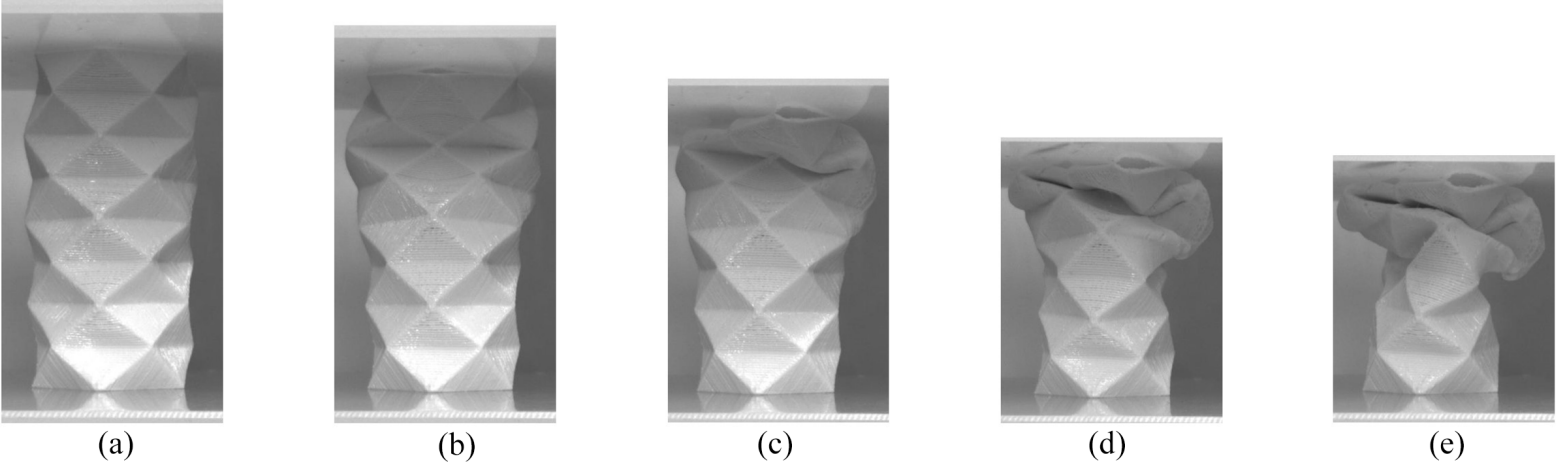
Compression progression of the flexible PLA Yoshimura tubular origami pattern. (a) The Yoshimura pattern before it started to buckle. (b) The Yoshimura pattern started to deform at the upper, horizontal, crease lines. (c) At 15.1 mm deformation, some of the folded creases started to curve. (d) As the tube continued to be compressed, buckling in the origami faces was also visible. (e) As the deformation continued to be increased, at 30.1 mm deformation the faces continued to curve and more buckling was visible around crease lines.

The Yoshimura pattern, as seen in Figure 9, did not fold along the origami crease lines during compression testing as uniformly as the accordion and Kresling patterns. The Supplemental Information Video S3 also shows how the Yoshimura pattern did not fold at crease lines. Figure 9(a) shows the Yoshimura origami tube at a deformation of 7.5 mm, when the peak force in the force-deformation curve occurred. After this point in the test, the tubular structure started to buckle, and once the buckling began, the force required to continue compressing the tube started to decrease. In Figure 9(b), the deformation in the tube occurred through the folding of a horizontal crease at the top of the tube. At 15.1 mm deformation in Figure 9(c), some of the crease areas that were folded started to curve. The deformation in the creases did not occur uniformly in the rows and some of the origami faces were starting to curve. The curvature and deformation in the origami faces becomes more apparent in Figure 9(d) and (e). The deformation in the Yoshimura patterned tube was less predictable than the accordion and Kresling patterned tubes tested in this study, which mostly deformed through folding of the origami creases.

The buckling behaviour observed during the compression test of the Yoshimura pattern could be due to other factors, such as the thickness of the tube and the angle between the panels. However, it is most likely that the increase in the overall mechanical property of the Yoshimura pattern is accounted for by the buckling effect. As shown in Figure 6(c), the force versus deformation curve for the Yoshimura sample reached all the way to 450 N before dropping, indicating that the structure is very good for load supporting but not ideal for absorbing compression. Additionally, Figure 9(b) revealed that the deformation caused expansion within the tubular structure itself, which is likely due to the complexity of the pattern, including the thickness of the tube and the angle between the panels, and could lead to buckling of the whole structure. The deformation in the Yoshimura patterned tube was less predictable than the accordion and Kresling patterned tubes tested in this study, which mostly deformed through folding of the origami creases. This is evident in Figure 9, where the curvature and deformation in the origami faces become more apparent in Figure 9(d) and (e).

Overall, the three different origami-inspired tubular patterns had distinctly different force versus deformation responses. The Yoshimura-patterned tube was able to withstand the highest forces. This is due to the methods in which the flexible origami-inspired tubes deformed. The deformation in the accordion and Kresling patterned tubes was most prominently observed through folding occurring at crease lines, showing that the forces were concentrated at these locations. The Yoshimura pattern primarily deformed through buckling rather than folding, as seen in the images in Figure 9. The buckling initially occurred at crease areas, however the areas surrounding the creases simultaneously buckled and deformed. This shows that while the crease areas did act to concentrate forces and cause the initial deformation, the pattern did not fold and deform in a predictable manner. This caused the Yoshimura patterned tube to be most resistant to compression.

The accordion and Kresling patterned tubes showed more predictability in their deformation methods. They both deformed through folding at crease lines and were similar in that the outer rows of the origami patterns deformed and folded first. As various rows folded, the force-deformation curve showed peaks and valleys. The peak forced occurred at the onset of folding in the rows, and the valley points occurred as a given row finished folding and more force was required to fold subsequent origami rows. The accordion pattern was the least resistant to compression loads since it folded in pure compression, whereas the Kresling pattern compressed and rotated internally when the patterned tube was folded during testing.

### 3.3. Activation results of SMP origami tubes

**Figure 10.**
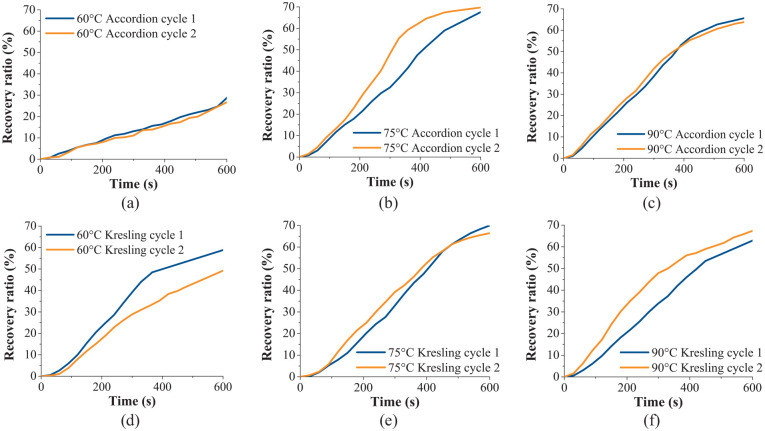
Change in shape recovery ratio of the SMP heat-activated origami-inspired tubes over time. The percent recovery of the SMP at 600 s is as follows: (a) cycle 1 – 29%; cycle 2 – 27%. (b) cycle 1 – 68%; cycle 2 – 70%. (c) cycle 1 – 66%; cycle 2 – 64%. (d) cycle 1 – 59%; cycle 2 – 49%. (e) cycle 1 – 70%; cycle 2 – 66%. (f) cycle 1 – 63%; cycle 2 – 67%.

Activation at three different temperatures was attempted for all three origami-inspired tubular patterns. During the programming stage of the Yoshimura sample, the tubular structure buckled, just as it had during the quasi-static compression testing, and fractured at all three temperatures. Hence, results for the shape memory polymer (SMP) activation of the tubes were only obtained for the accordion and Kresling patterns which folded, as expected, along their crease lines. The performance of the origami tubes was characterized using the shape recovery ratio (*R_r_*) as defined in equation (2):

(2) $$R_{r}=\frac{h_{t}-h_{i}}{h_{o}-h_{i}}$$

where *h_t_* was the height of the tube at the given recovery time, *h_i_* was height of the tube at the programmed height and *h_o_* was the original height of the tube. The shape recovery ratio of the accordion and Kresling samples over time at the three activation temperatures can be seen in Figure 10. For both patterns, the 60°C activation was the least effective with the smallest change over time during activation. It has been previously reported that lower programming temperature can lead to fractured cross-linking in a SMP, which negatively affects the programming shape and recovery of a SMP (Huang et al., 2012; Wang et al., 2020). This accounts for the 60°C activation of both tubular patterns having relatively lower shape recovery ratios compared to the results at the other tested temperatures. It is important to note that for all recovery observed, the origami patterned samples were initially folded to their maximum amount at the fold lines, where the origami faces came in contact with its adjacent faces.

As observed from the DSC results (Table 1), the cold crystallization temperature is around 95°C. To avoid the possible crystallization during the heating process, the three chosen programming and activation temperatures were all below the 95°C. The three activation temperatures, 60°C, 75°C and 90°C, were chosen to be above the glass transition temperature and below the cold crystallization temperature. The lowest temperature was about 5°C above the glass transition temperature and the highest activation temperature was about 5°C below the crystallization temperature, with the middle temperature chosen to be half-way between the lowest and highest chosen temperatures. This provided a range that was ideally suited for activation and provided an opportunity to determine if there were any effects on changing the programming and activating temperature. The shape recovery ratios shown in Figure 10 demonstrate that the programming and activation at 75°C and 90°C were similar. This shows that there was no evidence of rigidity due to crystallization.

At the beginning of the activation cycle, the recovery ratio increases relatively slowly. The tubular structures have thicknesses of 2 mm, therefore the initial slow activation occurs as the room-temperature tubes start to heat to the temperature inside the oven. Looking at the results for the tests performed at 75°C and 90°C, after the initial heating phase with slow recovery, the recovery ratio starts to increase at a steady pace. Near the end of the test, as the sample approaches 600 s of activation time, the rate of the shape recovery ratio starts to decrease as the origami tubular structures approach their maximum recovery height.

**Figure 11.**
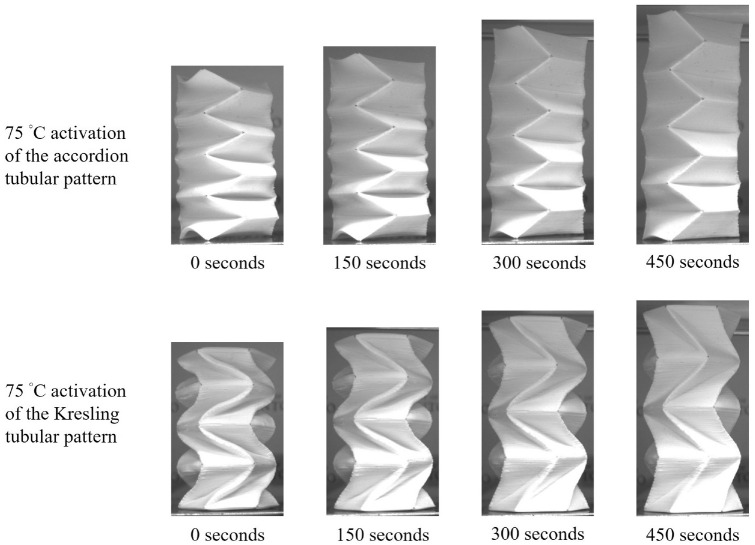
Images of the second cycle of the SMP at 75°C for the accordion and Kresling origami tubes during heat activation.

The shape recovery ratios of the accordion tube tested at 75°C were higher than the accordion pattern tested at 90°C for both cycles of the test. Images of the recovery of the accordion pattern during the second cycle at 75°C can be seen in Figure 11, and videos of the activation can be found in the Supplemental Information Video S4. At 150–300 s the top unit cells of the accordion pattern appear to have unfolded more than the bottom units. At the 60°C activation temperature, the bottom unit cells of the origami still appear to be folded more than the top of the tube at the end of the test, which accounts for the low recovery ratio at this temperature. When the accordion tubes are activated at 75°C and 90°C, the bottom unit cells unfold during the second half of the activation so that the bottom and top unit cells of the origami are unfolded to a similar degree. The reason that the top cells unfold before the bottom cells is that there is less resistance at the top of the origami tube. The weight of the upper unit cells of the origami tube applies a load to the bottom unit cells that must be overcome during activation.

The trend of the upper unit cells unfolding before the bottom cells seen in the accordion pattern was visible in the Kresling pattern at the activation temperature of 60°C. At the 75°C and 90°C activation temperatures, the final recovery ratios at the end of the test were higher than for the 60°C activation. This is consistent with the results from the accordion pattern. Overall, 60°C, although higher than the glass transition temperature, is the least effective activation temperature tested. At the 75°C activation temperature, the bottom and top origami rows of the Kresling pattern unfolded uniformly, as can be seen in Figure 11 and in the Supplemental Information Video S5. When the Kresling pattern folds and unfolds, there is a twisting motion in the tube. This twisting motion would apply an internal torque during activation which may have helped overcome the self-weight load that the upper unit cells of the tube would apply to the lower unit cells. The activation at 90°C was less uniform than at 75°C. This could be due to the origami pattern cracking at the horizontal crease lines during the programming stage (see Supplemental Information Figure S4 for images of cracking at 90°C activation). While some small cracks in the horizontal creases can be seen in the sample tested at 75°C, they are significantly smaller than the cracks in the 90°C Kresling origami tube and there is no sign that the cracks at lower temperature are affecting the activation. Therefore, despite the shape recovery ratio for the Kresling pattern being similar at 75°C and 90°C activation, the 75°C activation performed better overall due to more uniform unfolding and less cracking in the sample during the programming phase of the shape memory cycle.

### 3.4. Potential application of soft SMP 4D printed tubes

Origami-inspired SMPs have the potential to be used as self-unfolding structures. Applications that could incorporate the use of self-unfolding origami and would especially benefit are devices that are required to be portable, such as rehabilitation devices. Current rehabilitation devices are manipulated using DC motors and cables, resistance springs, pneumatic actuators and shape memory alloys (Chen et al., 2019, 2020; Hassanin et al., 2017; Sarac et al., 2019). While devices made using these movement types are portable, they typically require excess structures. For example, a pneumatic actuator would need to be able to store pneumatic fluids, and cable-driven devices would need to store excess cable.

**Figure 12.**
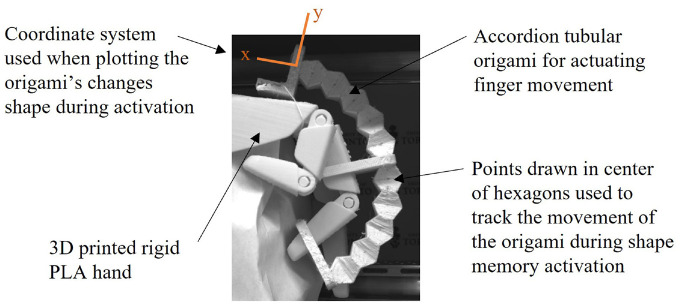
Test setup of finger rehabilitation prototype using SMP tubular self-unfolding origami.

A prototype of the self-unfolding origami application was built to move a finger on a model hand during SMP actuation. The change in shape was tracked along the centre of the origami using the stereovision setup. The triangulation code in the toolbox outputs the 3D coordinates of point locations with the camera acting as the origin point for the coordinate system. To graph and track the location changes, the coordinates of the point locations were transferred to the new coordinate system shown in Figure 12. This was done by translating and rotating the location coordinates that were calculated based on the camera’s location to determine the coordinates with respect to the new coordinate system.

Two activation cycles were performed, and it can be seen from the results that the programmed shape at 0 s of the second cycle was straighter than the first. The curved origami was difficult to grip and compress during the first programming phase of the experiment. The second programming step was less challenging. During the testing of the origami tubes, it was seen that cracks occurred during the SMP programming step, especially in the Kresling pattern. Although the accordion pattern did not exhibit the same large cracks at crease areas as the Kresling pattern, there was some plastic deformation at the crease locations when the pattern was folded due to the large strains incurred. Therefore, the first programming cycle of the finger heat activated unfolding origami prototype weakened the origami structure so that it was less resistant to folding during the second programming cycle, resulting in a straighter programmed configuration for the second actuation cycle.

**Figure 13.**
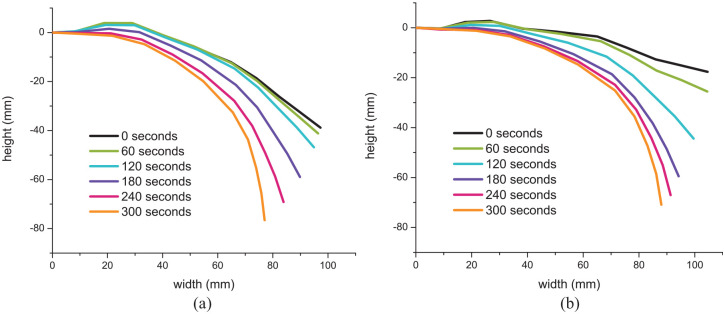
Movement tracking of the self-unfolding origami during activation in the rehabilitation prototype: (a) first activation cycle and (b) second activation cycle.

Comparing the curves of the first and second activation in Figure 13, the second actuation cycle experienced faster recovery at the beginning of the test. This could be due to the larger strains during the second programming. Overall, the centre portion of the test seemed to activate the fastest for both cycles, with the start and end of the test activating slower. This is consistent with the actuation of the tubes which also had slower recovery during the beginning and end of the tests. During the beginning of the tests, the origami unfolded slowly because they have just been introduced to their activation temperature and require time to thoroughly heat. At the end of the test, the origami shapes activate more slowly as their final geometries are approached.

**Figure 14.**
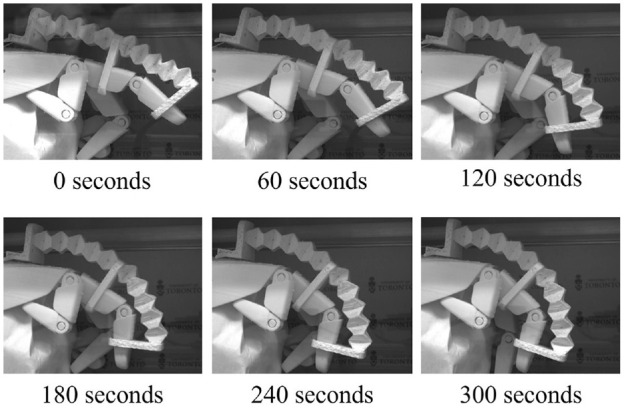
Images of prototype during second cycle of activation.

Images taken during the second activation of the finger rehabilitation prototype can be seen in Figure 14. A video of the prototype activation can be found in the Supplemental Information Video S6. The images show the progression of the finger movement as the origami unfolds, demonstrating that the origami SMP is effective in the finger-movement prototype. This proof-of-concept shows that the origami is capable of curvature, and is not limited to only linear motion. During the compression and shape memory activation of the Kresling pattern, the tube rotated. This means that for applications requiring curvature and rotation, perhaps both the accordion and Kresling designs could be incorporated to achieve a larger range of motion.

## 4. Conclusion

The three origami-inspired tubular patterns, when 4D printed using a flexible PLA filament, each had their own unique behaviours. The Yoshimura pattern was strongest according to the force-deformation graphs during the compression testing. However, this design did not fold along the crease lines as expected, and instead experienced buckling deformation when compressed. The Yoshimura pattern was therefore unsuitable for the heat activated unfolding SMP actuation since it did not fold predictably. Both the accordion and Kresling patterns folded along their crease lines during the compression testing, and their compressed, folded, configurations were successfully captured during the programming stage of the shape memory activation. The differences in deformation results can be attributed to the folding pattern of the Yoshimura structure, which enhances its overall mechanical properties and prevents compressive forces from traversing across the structure. In contrast, the accordion and Kresling patterns allow for compression along the folding pattern. The most effective activation temperature was found to be 75°C and the accordion design was the most effective pattern, mainly due to the Kresling pattern cracking at crease locations during shape programming. The accordion pattern was successfully used to design and activate a prototype device showing how the SMP, origami-inspired, tubular structure can be used in rehabilitation devices.

## Supplemental Material

sj-docx-1-jim-10.1177_1045389X231181940 – Supplemental material for 4D printed origami-inspired accordion, Kresling and Yoshimura tubes Click here for additional data file. Supplemental material, sj-docx-1-jim-10.1177_1045389X231181940 for 4D printed origami-inspired accordion, Kresling and Yoshimura tubes by Anastasia L Wickeler, Kyra McLellan, Yu-Chen Sun and Hani E. Naguib in Journal of Intelligent Material Systems and Structures
