# Supplementary material for: 4D printed origami-inspired accordion, Kresling and Yoshimura tubes
Source: J Intell Mater Syst Struct. 2023 Jun 21;34(20):2379–92. doi: 10.1177/1045389X231181940 (PMC10638089; doi:10.1177/1045389X231181940)
Supplement: sj-docx-1-jim-10.1177_1045389X231181940 – Supplemental material for 4D printed origami-inspired accordion, Kresling and Yoshimura tubes [file sj-docx-1-jim-10.1177_1045389X231181940.docx]

**Supplementary Information**

**4D Printed Origami-Inspired Accordion, Kresling, and Yoshimura Tubes**

Anastasia L. Wickeler^1^, Kyra McLellan^1^, Yu-Chen Sun^1^, and Hani E. Naguib *^1,2,3^

^1^ Department of Mechanical and Industrial Engineering, University of Toronto, 5 King's College Road, Toronto, ON, Canada, M5S 3G8

^2^ Department of Materials Science and Engineering, University of Toronto, Canada

^3^ Institute of Biomedical Engineering, University of Toronto, Canada

* Email: naguib@mie.utoronto.ca

Checkerboard pattern for digital image calibration

Oven for heating samples during shape memory activation


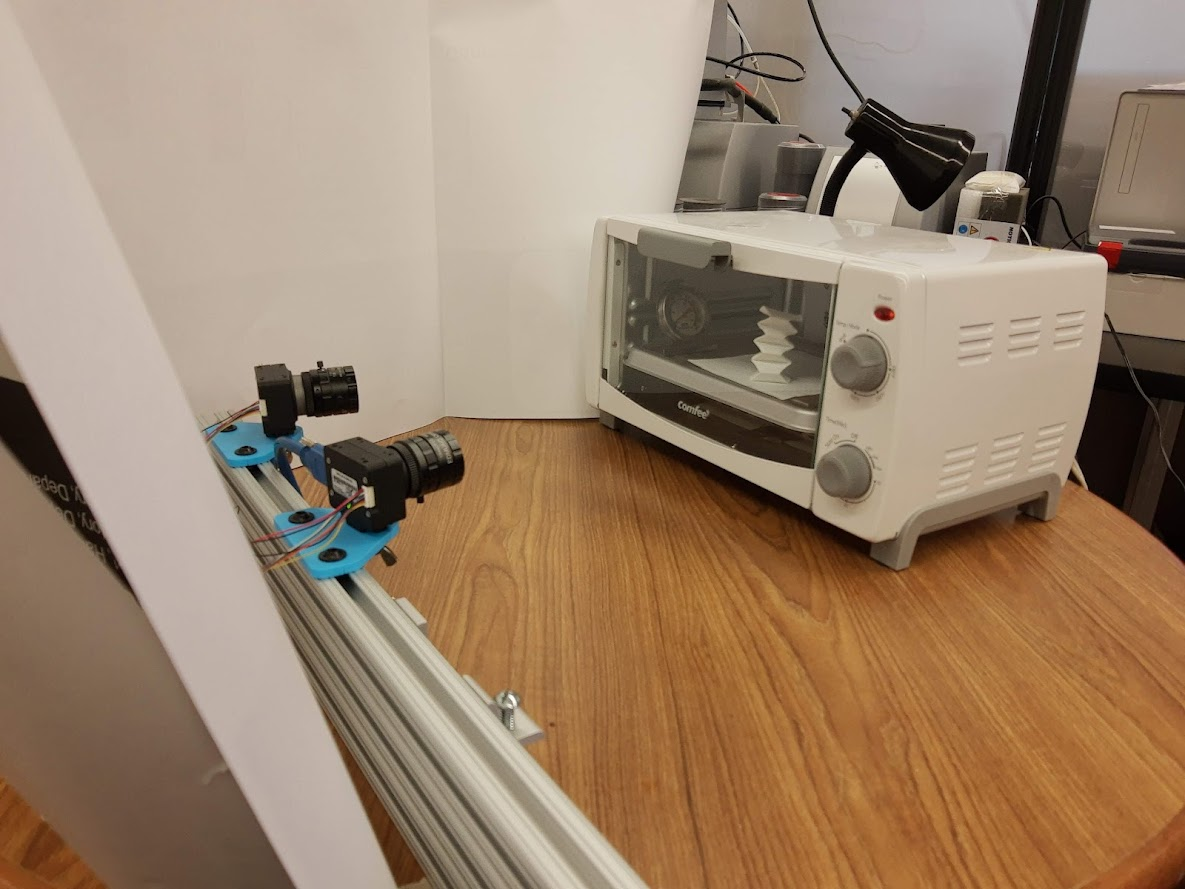

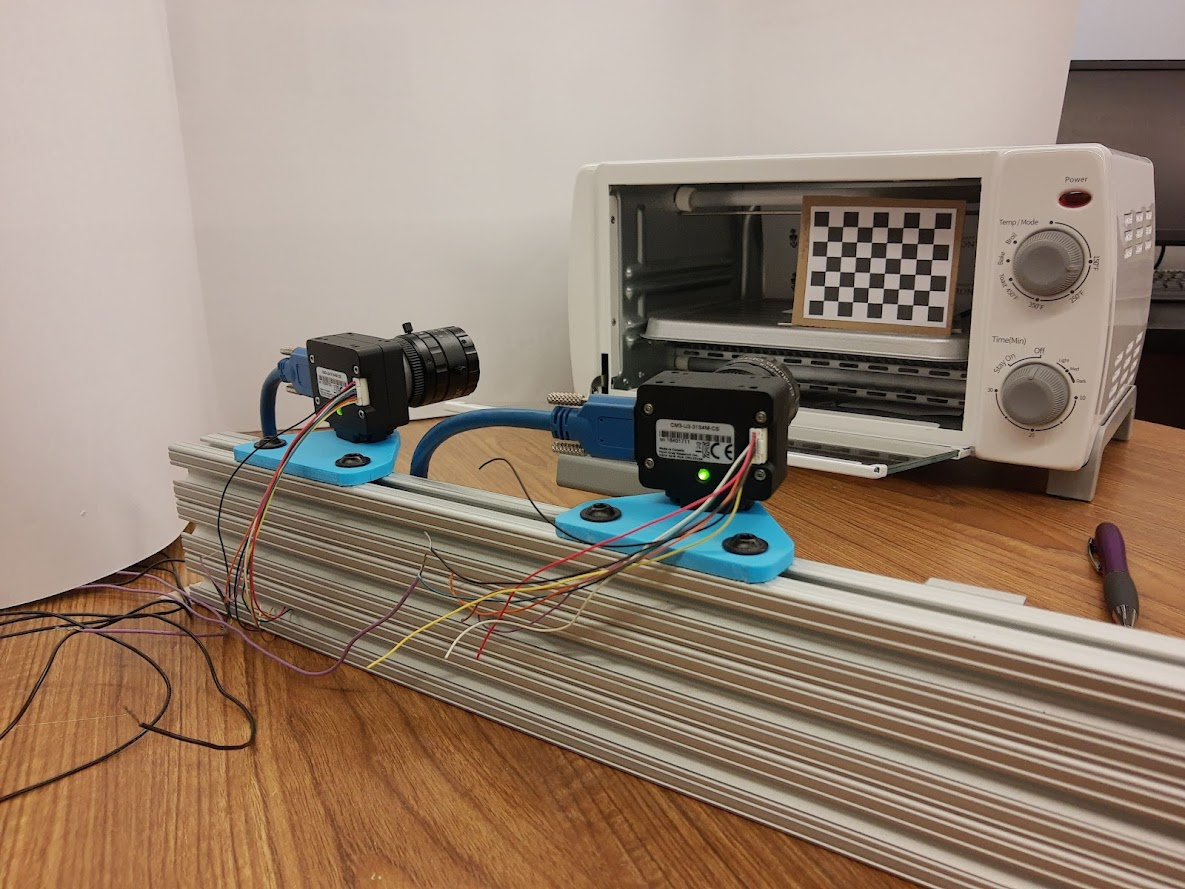


Left camera for stereo vision

Right camera for stereo vision

Shape memory sample being activated

S1. Stereo vision setup for digital image calibration (left) and for sample measurements during shape memory activation (right)

| 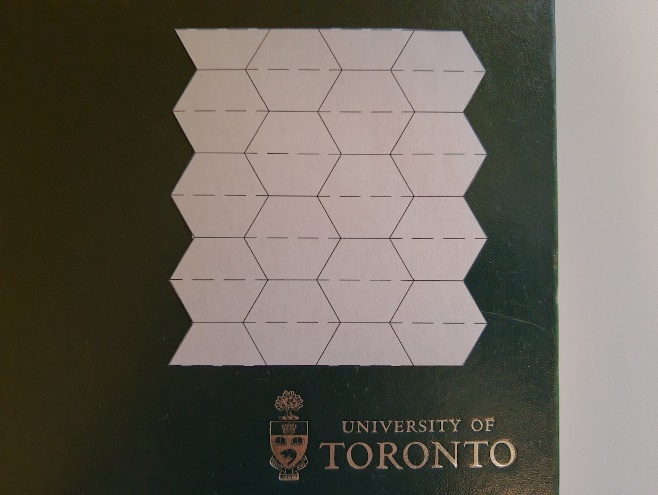  Unfolded accordion pattern | 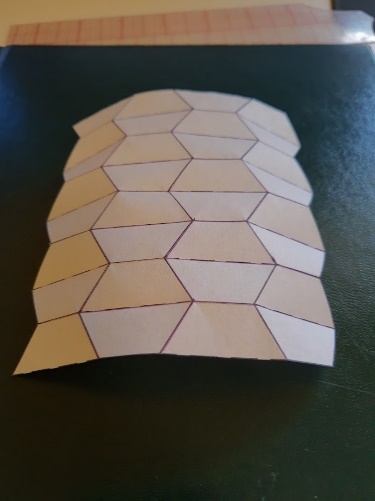  Partially folded accordion pattern | 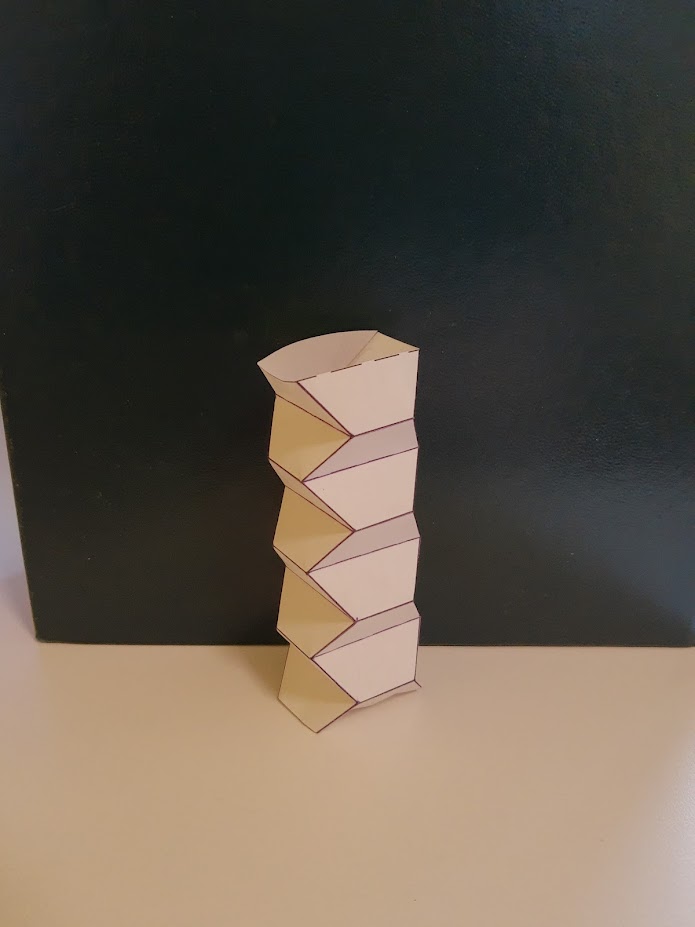  Fully folded accordion pattern |
| --- | --- | --- |
| 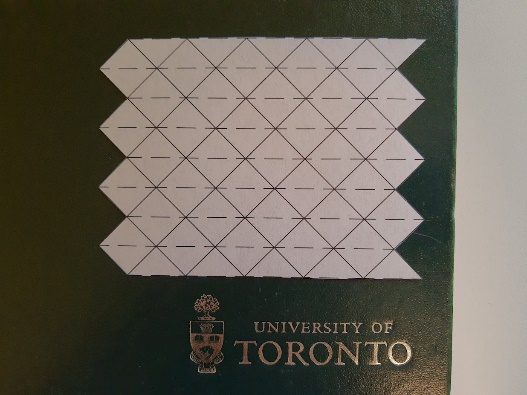  Unfolded Yoshimura pattern | 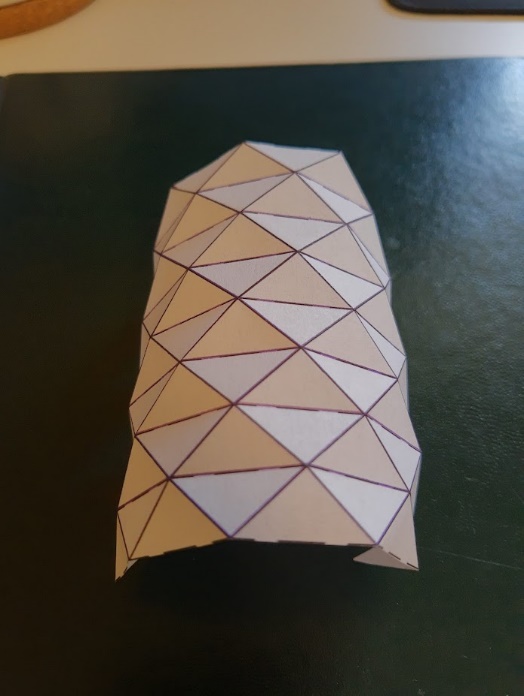  Partially folded Yoshimura pattern | 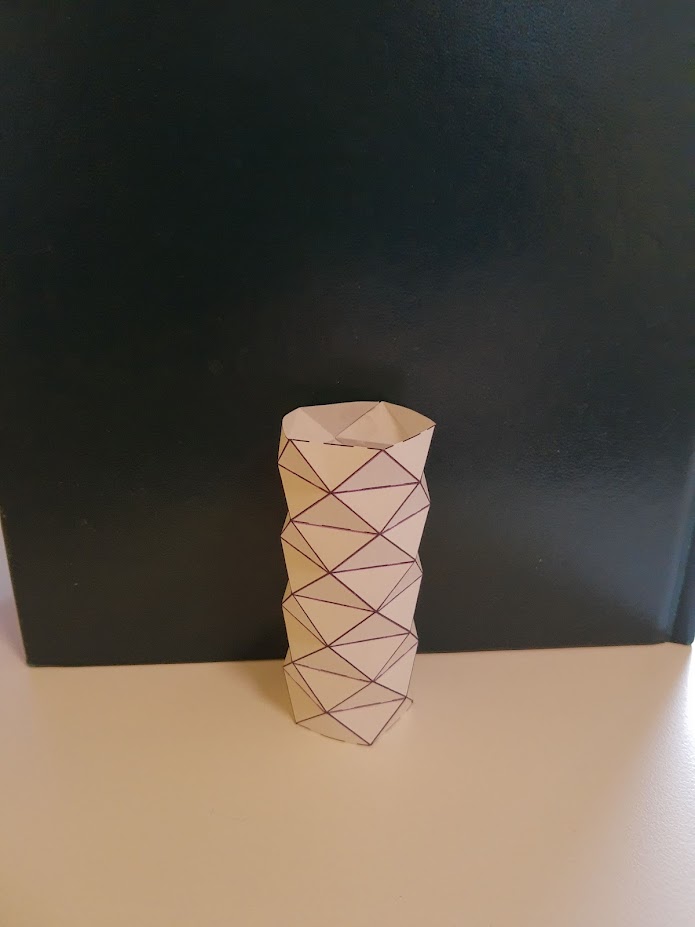  Fully folded Yoshimura pattern |
| 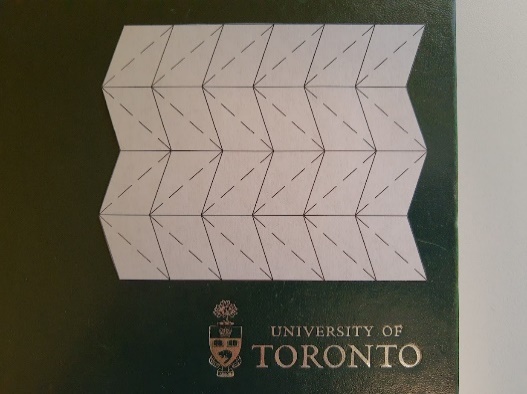  Unfolded Kresling pattern | 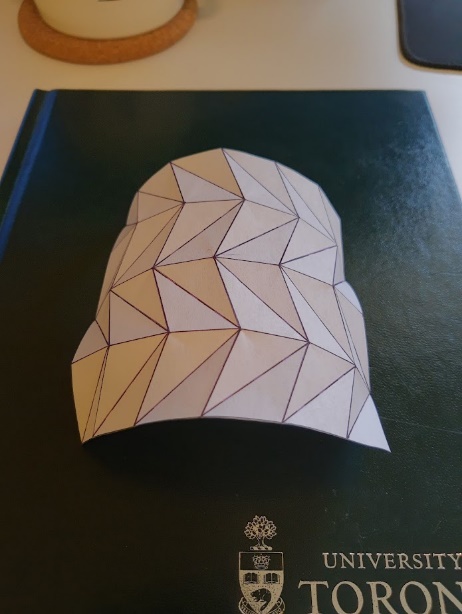  Partially folded Kresling pattern | 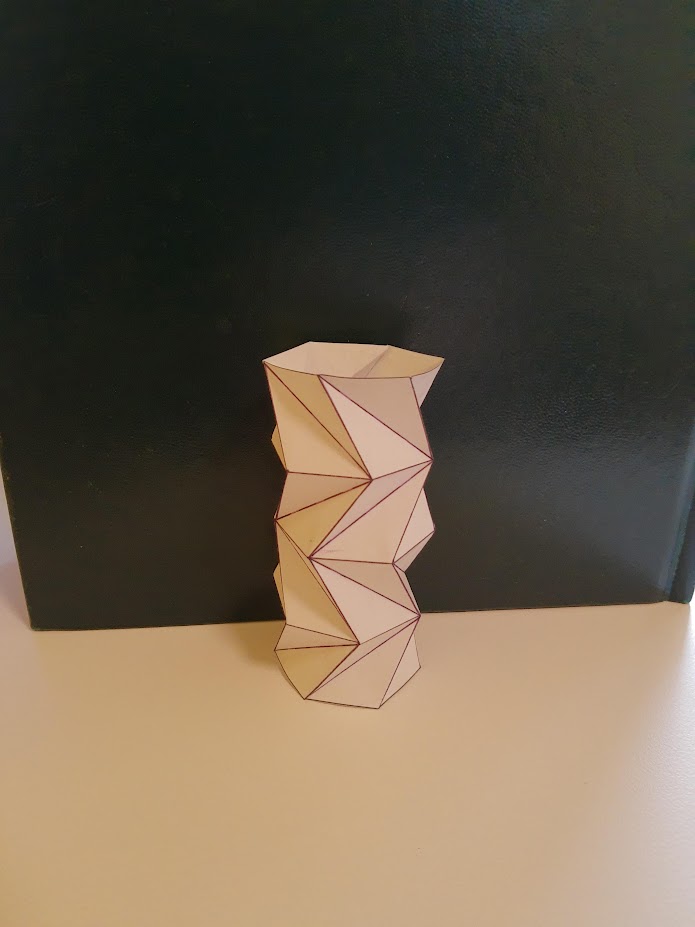  Fully folded Kresling pattern |

S2. Images of the folded origami used to inspire the 3D printed tubular designs. Images are shown of the pattern flat, partially folded, and fully folded for all three origami patterns. The patterns were scored using a pen during the folding process, therefore the dashed lines in the fold patterns no longer appear dashed once the patterns were folded.

Tubular sample for compression testing


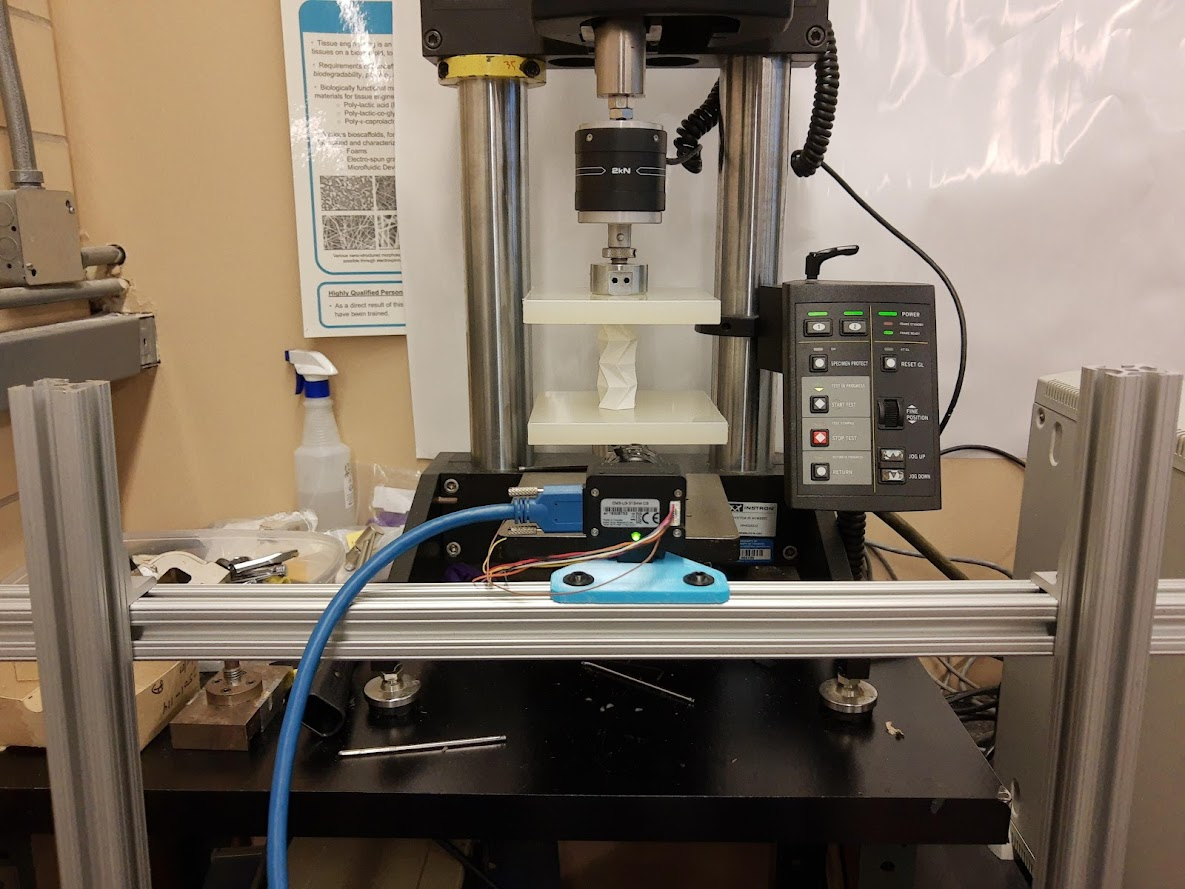


Load cell

Camera for recording compression test

S3. Compression test setup of tubular origami-inspired structures.

| 90 °C activation | 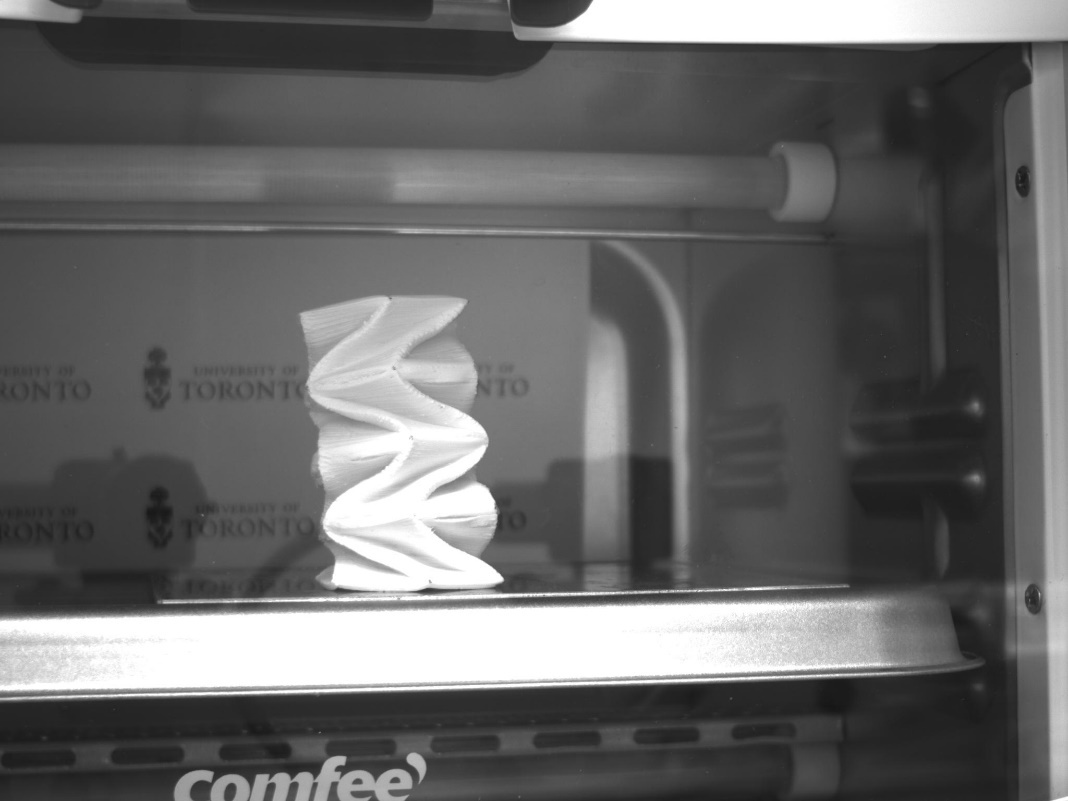  0 seconds | 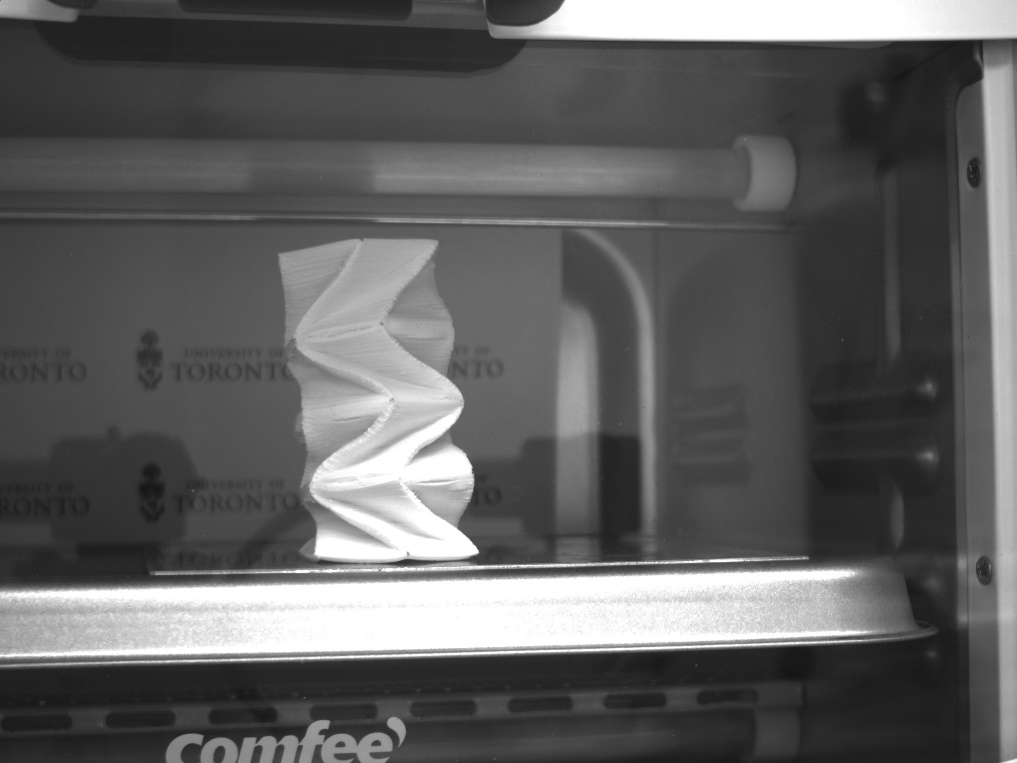  150 seconds | 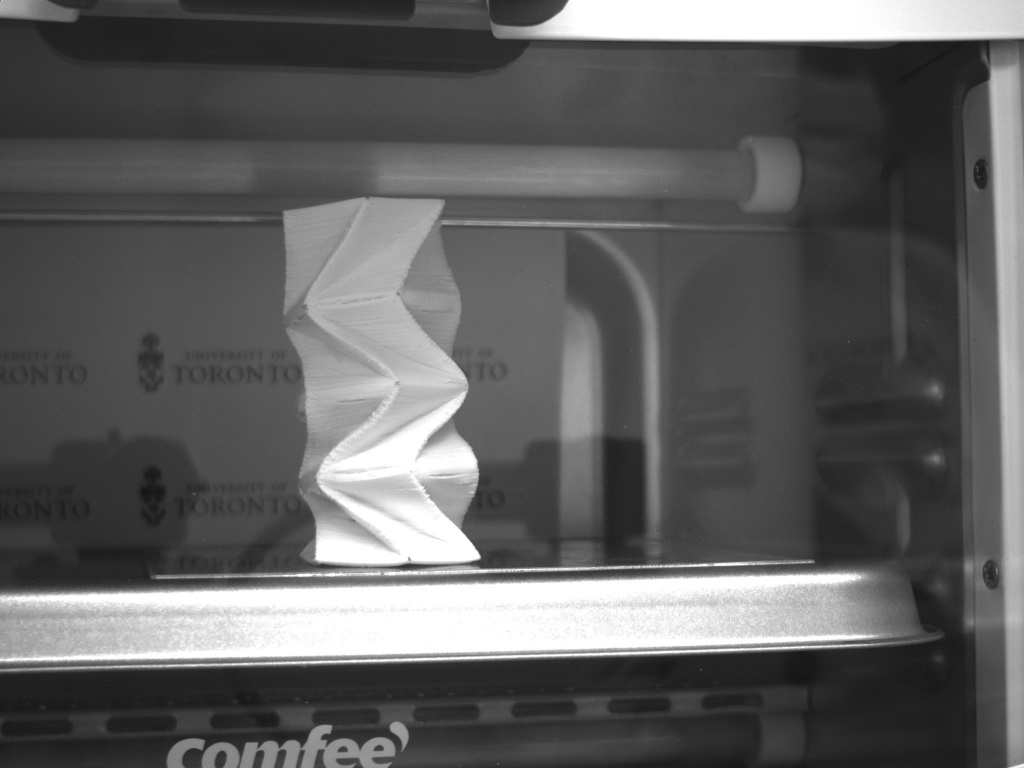  300 seconds | 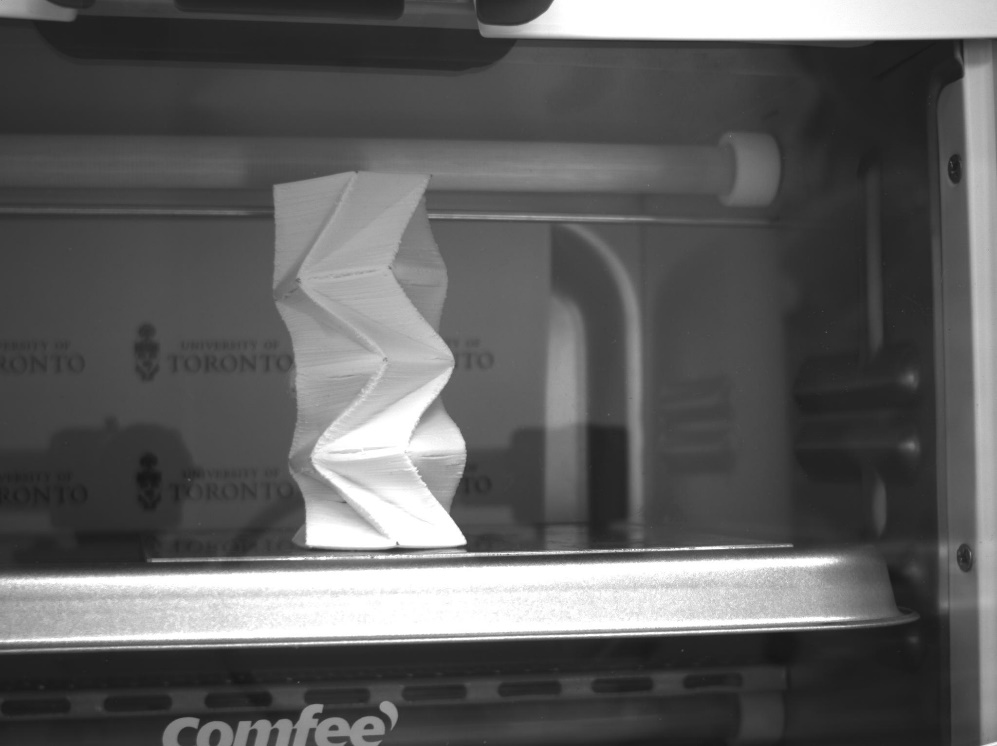  450 seconds | 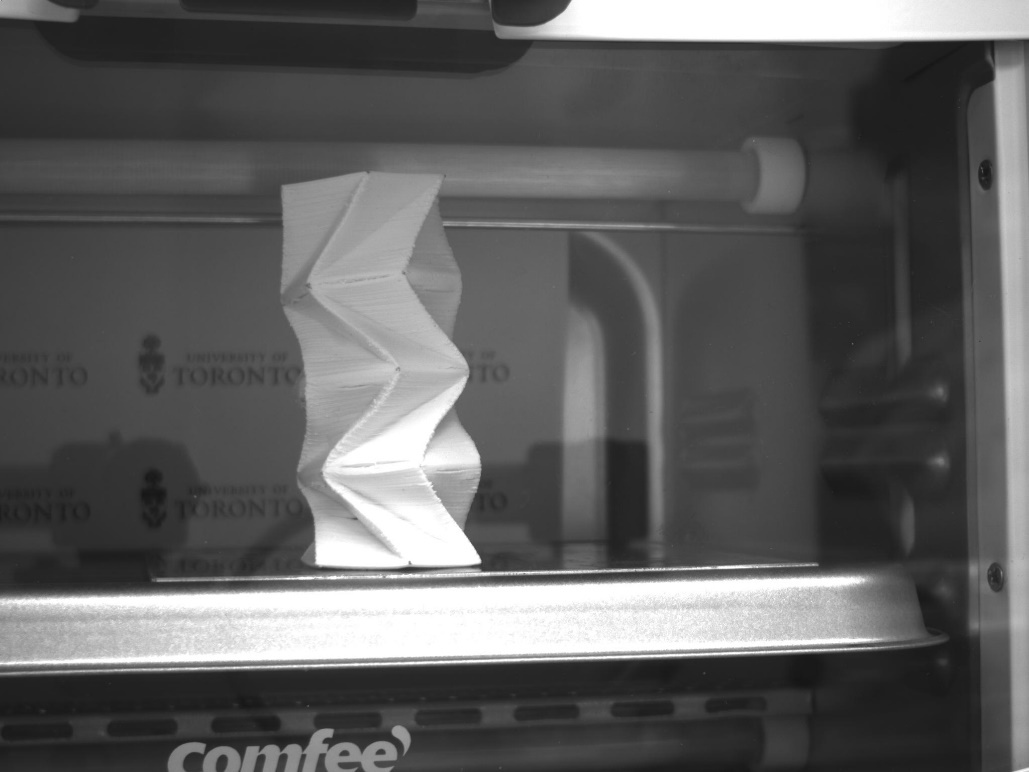  600 seconds |
| --- | --- | --- | --- | --- | --- |

S4. Images of the second cycle of the SMP Kresling origami tube at 90 °C activation temperature showing cracks at the horizontal crease lines.
